# Evaluating stool microbiome integrity after domestic freezer storage using whole-metagenome sequencing, genome assembly, and antimicrobial resistance gene analysis

**DOI:** 10.1128/spectrum.02278-24

**Published:** 2025-02-11

**Authors:** Paula Momo Cabrera, Nicholas A. Bokulich, Petra Zimmermann

**Affiliations:** 1 Department of Community Health, Faculty of Science and Medicine, University of Fribourg, Fribourg, Switzerland; 2 Laboratory of Food Systems Biotechnology, Department of Health Sciences and Technology, ETH Zurich, Zurich, Switzerland; 3 Department of Paediatrics, Fribourg Hospital, Fribourg, Switzerland; 4 Infectious Diseases Research Group, Murdoch Children’s Research Institute, Parkville, Australia; 5 Department of Paediatrics, The University of Melbourne, Parkville, Australia; The Pennsylvania State University Food Science, University Park, Pennsylvania, USA; University of Pennsylvania, Philadelphia, Pennsylvania, USA; Hvidovre Hospital, Hvidovre, Denmark

**Keywords:** microbiota, fecal, gut, infant, shotgun sequencing, −20°C

## Abstract

The gut microbiome is crucial for host health. Early childhood is a critical period for the development of a healthy gut microbiome, but it is particularly sensitive to external influences. Recent research has focused on using advanced techniques like shotgun metagenome sequencing to identify key microbial signatures and disruptions linked to disease. For accurate microbiome analysis, samples need to be collected and stored under specific conditions to preserve microbial integrity and composition, with −80°C storage considered the gold standard for stabilization. This study investigates the effect of domestic freezer storage on the microbial composition of stool samples obtained from 20 children under 4 years of age with the use of shotgun metagenome sequencing. Fresh stool samples were aliquoted into sterile tubes, with one aliquot stored at 4°C and analyzed within 24 hours, while others were frozen in domestic freezers (below −18°C) and analyzed after 1 week, 2 months, and 6 months. Assessments of contig assembly quality, microbial diversity, and antimicrobial resistance genes revealed no significant degradation or variation in microbial composition.

**IMPORTANCE:**

Most prior studies on sample storage have relied on amplicon sequencing, which is less applicable to metagenome sequencing—given considerations of contig quality and functional gene detection—and less reliable in representing microbial composition. Moreover, the effects of domestic freezer storage for at-home stool collection on microbiome profiles, contig quality, and antimicrobial resistance gene profiles have not been previously investigated. Our findings suggest that stool samples stored in domestic freezers for up to 6 months maintain the integrity of metagenomic data. These findings indicate that domestic freezer storage does not compromise the integrity or reproducibility of metagenomic data, offering a reliable and accessible alternative for temporary sample storage. This approach enhances the feasibility of large-scale at-home stool collection and citizen science projects, even those focused on the more easily perturbed early life microbiome. This advancement enables more inclusive research into the gut microbiome, enhancing our understanding of its role in human health.

## INTRODUCTION

The composition of the stool microbiome has been linked to many diseases, including allergic, inflammatory, rheumatological, metabolic, and psychiatric diseases (1–5). To accurately determine dysbiotic or suboptimal microbiome states and identify confounding factors, longitudinal sampling of large, representative population cohorts is essential. However, this can be logistically challenging. The current gold standard approach for preserving microbiome integrity is immediate DNA extraction or freezing of the stool sample at −80°C as the addition of stabilization buffers can affect DNA quantity and purity or lead to bacterial cell lysis (6, 7). While a number of studies have now tested refrigeration and room-temperature storage conditions (8–14), the impact of freezer temperature on microbiota composition during storage warrants further attention.

Immediate DNA extraction is not always feasible, particularly in studies including geographically dispersed participants. Studies using 16S rRNA amplicon sequencing methods show that DNA integrity and microbial composition are maintained with storage of the stool sample at room temperature for up to 24 hours (8, 15) , at 4°C for up to 24–72 hours (15–18), and at −20°C for up to 7 days (15, 18, 19). Conversely, other studies indicate that storage of the stool sample at room temperature over 12–72 hours (9, 16, 20) and at −20°C for 3–7 days (21) results in significant changes in the bacterial composition. One study has even reported changes in the relative abundance of bacterial phyla after just 30 minutes of exposure to room temperature (21). Recent studies suggest that storage of the stool sample at −20°C may suffice for short-term preservation (22), challenging the necessity of −80°C freezing. Thus, it remains uncertain whether the widely accepted gold standard of −80°C freezing is truly necessary, offering a possibility for investigating alternative cost-effective and accessible storage solutions.

A key concern with using domestic freezers is the occurrence of freeze−thaw cycles, particularly in frost-free freezers. These cycles cause periodic temperature fluctuations, which have been linked to changes in microbiome composition in some studies (19, 21), but not in others (18). These differences may reflect variability in sample sizes (*n*  =  11, 4, and 3) and study design. Most modern household refrigerator-freezers are equipped with automatic frost removal systems that operate in cycles lasting between 10 and 30 minutes (23, 24), typically occurring once or twice daily. During these defrost cycles, the air temperature in the freezer can increase to approximately −4°C (25), which could potentially impact the stability and preservation of the microbial community.

In this study, we investigated the stability of microbial composition in stool samples obtained from young children, which are stored in home freezers (−18°C) over 6 months, a condition that has not been previously investigated and could pose significant logistical advantages to current sample collection and storage methods.

Enabling participants to store samples in their home freezers can reduce the frequency of nurse visits for sample collection, thereby lowering personnel and travel costs, as well as the logistical burden on participants and study coordinators. This becomes particularly relevant as the field moves toward larger cohort sizes and higher temporal density in microbiome samples.

## RESULTS

### Stool microbial diversity

**Fig 1:**
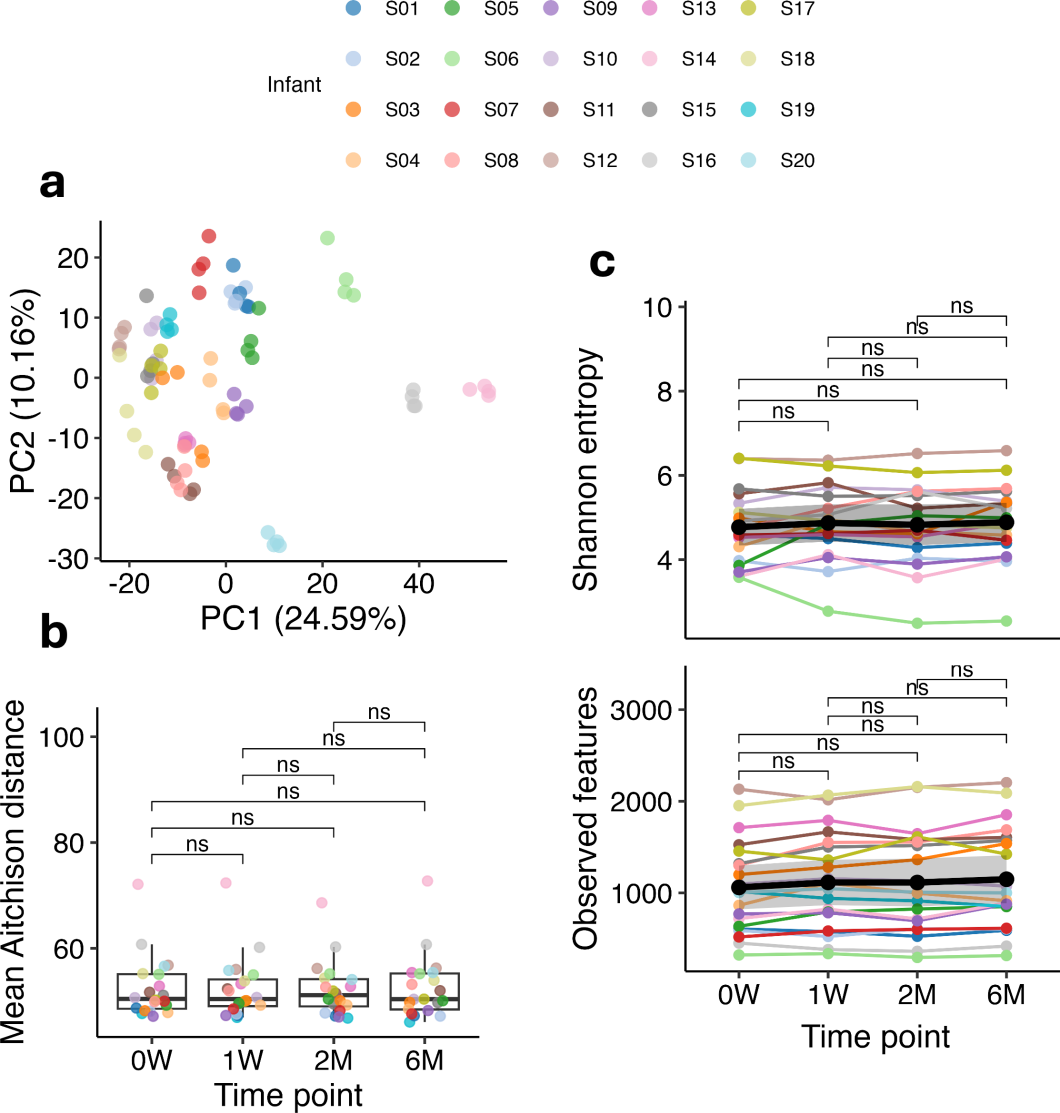
(**a**) PCoA based on the Aitchison distance matrix, illustrating the beta diversity of the microbial communities. Each dot represents a sample, colored by child ID (S01–S20). (**b**) Mean Aitchison distance between samples at different time points, representing compositional differences. The mean distance is calculated as the average pairwise Aitchison distance for all samples within each time point, with individual dots representing the pairwise distances between samples. (**c**) Alpha diversity metrics across time points: the Shannon diversity index (top panel) and observed features (bottom panel) are shown at 0 weeks (0W), 1 week (1W), 2 months (2M), and 6 months (6M). The Shannon index reflects diversity within samples, while observed features indicate species richness. Thick black lines represent the mean values, with shaded gray areas denoting standard deviation. The Wilcoxon rank-sum test with Bonferroni correction was used to assess differences, with no significant differences (ns) observed between the time points for panels b and c.

To investigate the stability of microbial diversity in stool samples from our cohort of 20 children stored in home freezers over a 6-month period, we analyzed read-based metagenomic data from samples collected at four distinct time points: week 0 (0W), week 1 (1W), month 2 (2M), and month 6 (6M). Principal component analysis on Aitchison distances (Fig. 1a) indicated that storage at −20°C had a minimal impact on the overall microbial community structure as no clustering by storage duration was observed. The mean Aitchison distance, which represents compositional differences between samples at different time points, showed no significant variations (Fig. 1b). Bray−Curtis and Jaccard distance metrics (Fig. S1a and b respectively), further showed that inter-individual variability surpasses temporal variability. As illustrated in Fig. 1c, alpha diversity remained stable over time, with no significant differences observed between time points, not even in comparison to the unfrozen samples at 0W. Additionally, the number of observed species (Fig. 1c) showed no significant differences across time points.

### Inter-individual differences have a greater influence on stool microbial diversity than temporal effects

To better understand the factors influencing the microbial community structure, we employed linear mixed effects (LME) models (Table S1). Our analysis revealed that storage time did not significantly affect microbial community composition when evaluated using Aitchison (*P* = 0.267, β = −0.03) and Jaccard (*P* = 0.836, β = 0.000) metrics, although a weak but significant effect was observed with Bray−Curtis (*P* = 0.007, β = −0.004).

In contrast, children’s age emerged as a significant factor, consistently reflecting inter-individual variation in microbial communities. This was particularly evident in the Aitchison (*P* = 0.005, β = 3.062) and Jaccard (*P* = 0.004, β = 0.019) metrics, although the Bray−Curtis metric did not show a significant effect (*P* = 0.629, β = 0.006).

Further supporting the minimal impact of temporal changes, PERMANOVA results indicated no significant differences in beta diversity across different time points (*P* = 1 for Aitchison, *P* = 0.935 for Bray−Curtis, and *P* = 0.992 for Jaccard) (Table S2).

To evaluate the relative contributions of temporal versus inter-individual variation in stool microbiome profiles, we implemented a random forest classifier to predict the sampling time points. The classifier’s performance was suboptimal, as evidenced by the confusion matrix (Fig. S2a), where no clear pattern of accurate classification emerged. The ROC curves (Fig. S2b) further illustrate the classifier’s inefficacy, with area under the curve (AUC) values across different time points ranging from 0.10 to 0.24, only marginally above the expected performance of random guessing. Notably, the overall accuracy failed to exceed random chance, emphasizing the difficulty in distinguishing samples based solely on their microbiome profiles over time (Fig. S2c).

### Temporal dynamics of specific taxa

**Fig 2:**
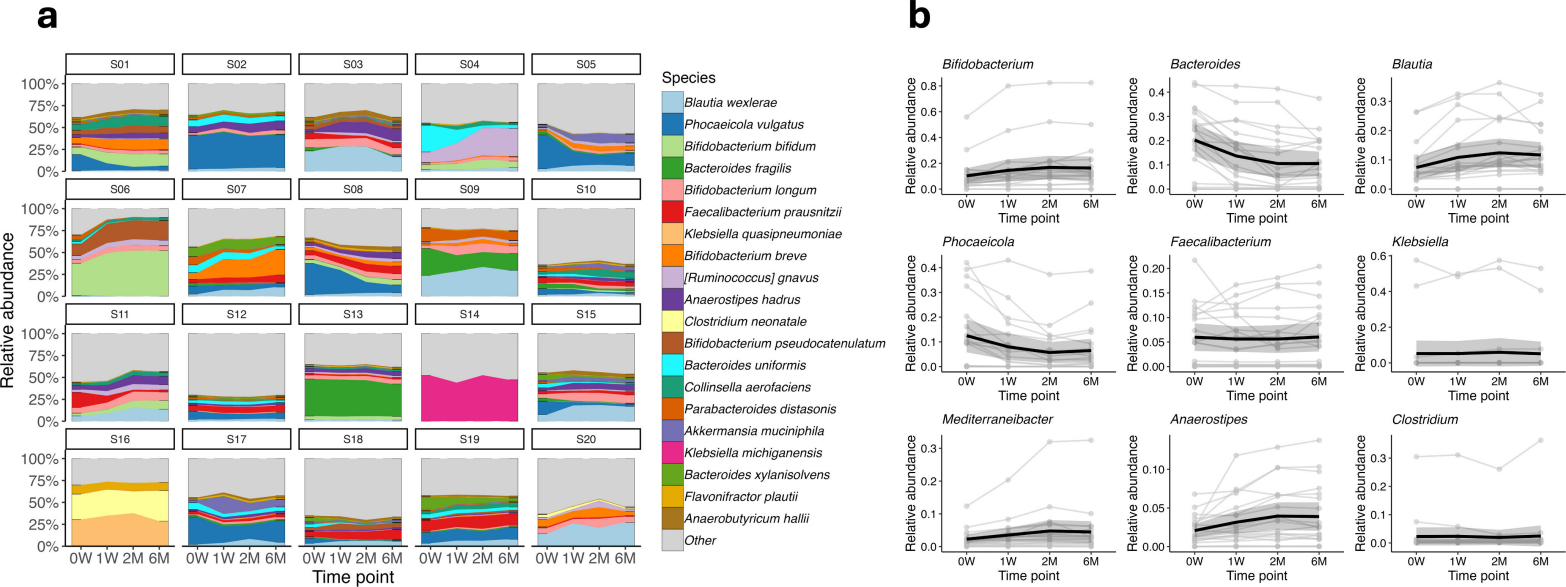
(**a**) Stacked area plots showing the relative abundance of the top 20 most abundant species across different time points (0W = week 0, 1W = week 1, 2M = month 2, and 6M = month 6) for each child (S01–S20). Each color represents a different species, illustrating the dynamic changes and stability in the microbial community composition over the first 6 months of life. (**b**) Line plots depicting the relative abundance trends of the most abundant genera. Each plot shows the relative abundance over time per individual child data (thin lines) and the overall mean trend (thick line).

A total of 115 species were detected at or above 1% relative abundance among all samples; however, none showed significant deviations from the pre-freezing baseline (0W) following ANCOM-BC analysis. As can be observed in Fig. 2, subtle qualitative fluctuations were noted among subjects over time, and these changes in the microbial community did not reach statistical significance neither at the genus or species level.

### Temporal variability in antimicrobial resistance

The longitudinal stability of AMR genes detected at 0W was assessed using AMRFinderPlus and RGI annotations (Fig. S3). Both tools identified clinically relevant AMR genes. Most genes detected at 0W remained consistently present across 1W, 2M, and 6M, demonstrating robust preservation under varying storage conditions.

Genes such as *tet(Q*) were consistently detected across all time points by both tools, highlighting their agreement in tracking highly prevalent AMR genes. However, some differences were observed between the tools: AMRFinderPlus focused on clinically significant genes and resistance mechanisms (e.g., *erm(B*) and *cfxA*), whereas RGI annotated a broader range of resistance genes, including those associated with efflux pumps (e.g., *mdtA* and *acrB*).

**Fig 3:**
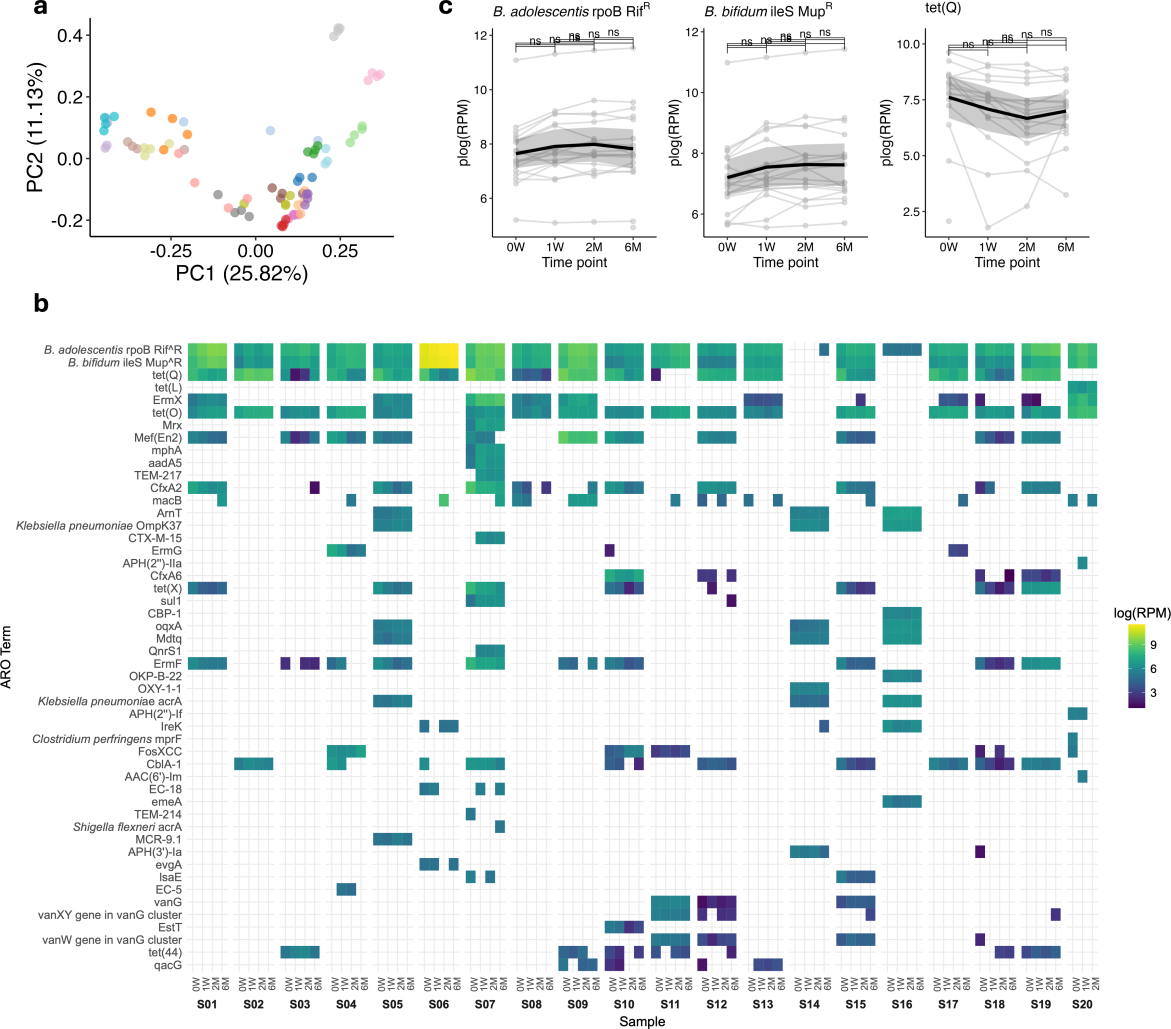
(**a**) Principal coordinate analysis (PCoA) plot depicting beta diversity based on the Jaccard distance matrix, calculated from antimicrobial resistance (AMR)-conferring genes. Each point represents a sample, colored by child (S01–S20), as indicated in the legend, with the percentage of variance explained by PC1 and PC2. (**b**) Heatmap of the top 20 most abundant ARO terms, displaying log-transformed normalized reads per million (RPM) values. (**c**) Line plots depicting the relative abundance trends of the most abundant AMR-conferring genes. Each plot shows RPM over time per individual child data (thin lines) and the overall mean trend (thick line). The y-axis is displayed on a pseudo-log scale (plog, log(1 + x)) to enhance visualization of small or zero values. The Wilcoxon rank-sum test with Bonferroni correction was used to assess differences, with no significant differences (ns) observed between the time points (0W = week 0; 1W = week 1; 2M = month 2; 6M = month 6).

To further explore AMR gene dynamics, changes in gene abundance were examined using read-based alignment to the CARD database via the RGI application. In Fig. 3a, the PCoA based on the Aitchison distance shows variability in AMR profiles across different subjects and time points. The first principal component (PC1) accounts for 25.82% of the variance, while the second principal component (PC2) explains 11.13%. To further understand the drivers of these observed changes, we analyzed the metadata variables contributing to the principal coordinates from the PCoA of Jaccard distance (Fig. S4). Specifically, time point (week) (R²=0.04 for PC1 and R²=0.013 for PC2) was demonstrated to be poorly correlated, consistently revealing the lack of impact of sample collection time points on AMR profiles. Conversely, child age (months) was the most substantial contributor to the observed variability (R²=0.54 for PC1 and R²=0.32 for PC2).

Furthermore, Fig. 3b displays antimicrobial resistance profiles over time. The analysis highlighted that the most prevalent AMR-conferring genes detected, including “B. adolescentis rpoB Rif^R” (mutated RNA polymerase β subunit (*rpoB*) conferring resistance to rifampicin) and “B. bifidum ileS Mup^R” (mutated isoleucyl-tRNA synthetase gene (*ileS*), conferring resistance to mupirocin), showed stable mean abundance overtime across all children. In contrast, “tet(Q)” (tetracycline-resistant ribosomal protection protein gene) showed a trend of decreasing abundance over time (Fig. 3c). However, none of this fluctuations resulted to be statistically significant (Fig. 3d).

### Contig assembly quality over time

Next, we evaluated the stability of contig assembly quality in stool microbiome samples over time to assess whether domestic freezer storage could induce DNA damage or other changes impacting assembly quality. We assessed key metrics such as N50 and L50 across four different time points: week 0 (0W), week 1 (1W), month 2 (2M), and month 6 (6M). These metrics are critical indicators of the quality and completeness of genome assemblies obtained from metagenomic sequencing data, representing the length of the shortest contig at the 50% genome assembly threshold (N50) and the number of contigs whose lengths sum to 50% of the genome assembly (L50).

**Fig 4:**
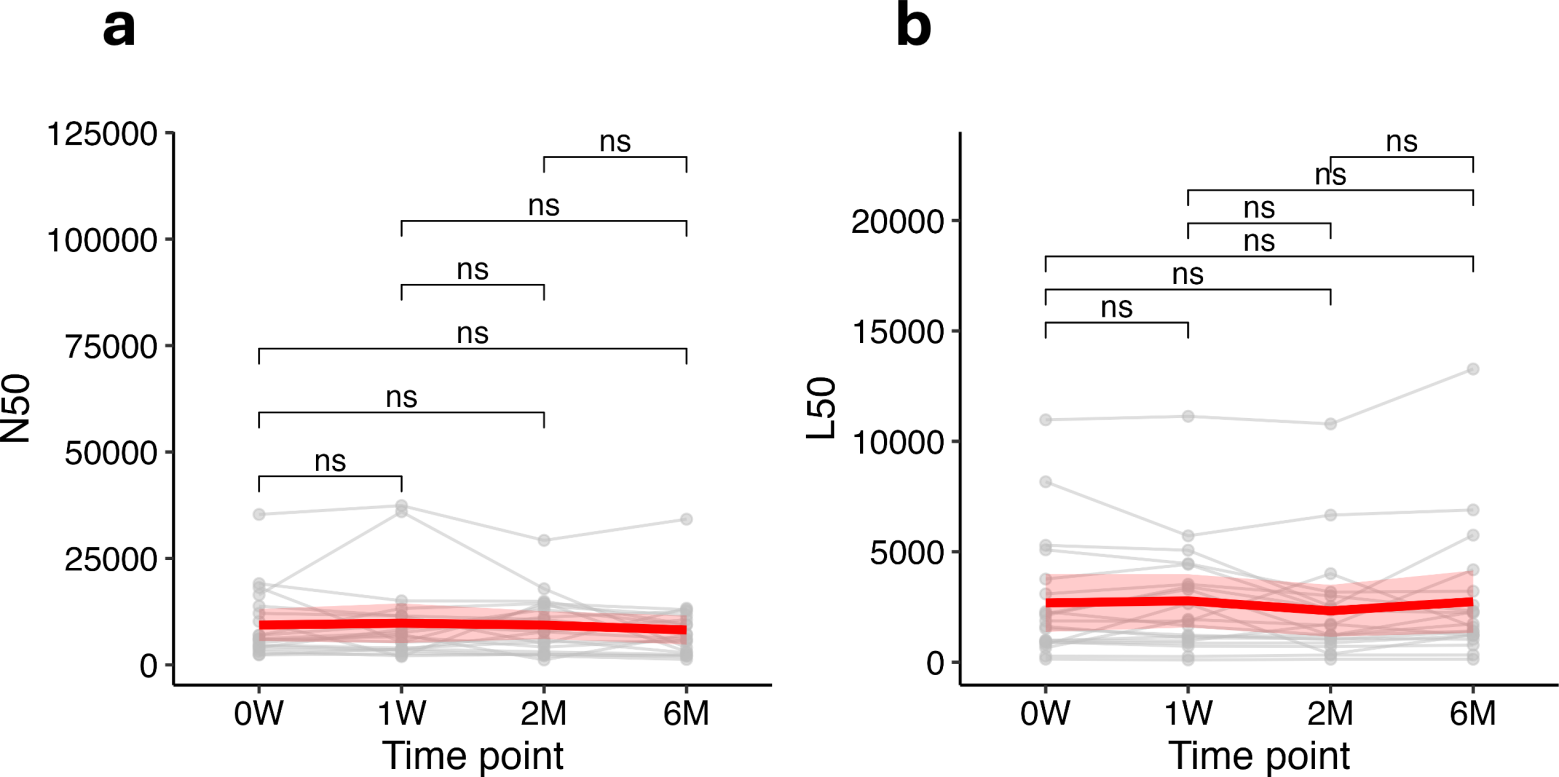
N50 (**a**) and L50 (**b**) metrics in stool microbiome samples stored over time (0W = week 0, 1W = week 1, 2M = month 2, and 6M = month 6). Line plots show individual child data (gray lines) and the overall mean trend (red line), with shaded red areas representing the standard deviation. The Wilcoxon rank-sum test with Bonferroni correction was used to compare metrics across time points, with no significant differences (ns) observed.

The median N50 values show slight fluctuations across the different time points, with no significant differences observed (ns) between 0W, 1W, 2M, and 6M. (Fig. 4a). Similarly, the L50 values across the same time points also exhibited minimal variation, with no significant differences (ns) detected between the different storage durations. (Fig. 4b).

## DISCUSSION

In the rapidly evolving microbiome field, efforts have increasingly been directed toward larger cohort sizes and higher temporal density in sample collection. In this context, the need for reliable, cost-efficient, and low-burden sample collection and storage solutions have become paramount. Ensuring that storage conditions do not introduce significant biases or artifacts in metagenome sequence data is crucial for accurately tracking functional gene composition and microbial community dynamics over time.

Our findings demonstrate that DNA integrity, microbial and resistance-conferring gene diversity, as well as contig assembly quality remain stable over a 6-month storage period in home freezers. By employing shotgun metagenomic sequencing, we achieved species-level resolution in our analysis, allowing for a more comprehensive assessment of microbial diversity that extends beyond the conventional focus on taxonomic stability during storage. This provides a holistic view of stool DNA stability under storage in home freezers, offering valuable insights for optimizing long-term storage protocols. Additionally, the study’s design, which included multiple time points, adds robustness to our findings.

In this study, the core microbiome retains its richness and evenness over time, irrespective of the increasing number of freeze−thaw cycles endured during storage in home freezers. While contig assembly results were an essential quality check, our study used read-based analyses, such as microbial diversity and AMR profiling, which provide more detailed insights into the microbial community structure and function. While the relative abundances of species and AMR genes may fluctuate with storage in home freezers, the overall community structure remains largely stable. In fact, similarly to what was observed by Ilett *et al*. (26), our study found no significant differences between non-frozen 0 W samples and the frozen 1W up to 6 M samples. However, specific microbial taxa which were found to have subtle temporal fluctuations in abundance included *Bacteroides* spp., whose relative abundance decreased, and *Bifidobacterium* spp., the relative abundance of which increased over the 6-month storage period. These taxa-dependent variations are consistent with previous studies and could be attributed to differences in bacterial cell wall structure, metabolic activity, and resilience to freezing and thawing processes (22, 27–29). These findings highlighted the intricate dynamics within microbial communities and the importance of considering taxa-specific responses when interpreting microbiome data. Our study identified both high stability in the most abundant and frequent resistance-conferring genes and subtle shifts in those less abundant and highly infrequent. These shifts are likely more related to the heterogeneity of the samples than to storage conditions, emphasizing the complex and dynamic nature of microbial communities. The prevalence of highly infrequent and sparse AMR-conferring genes suggest that while some resistance-conferring genes maintain a consistent presence, others appear to be more sporadic and sparse. These findings aligned with those of previous research (27), which demonstrated that different storage methods could influence the detection and quantification of AMR-conferring genes, impacting the interpretation of resistance profiles in microbial communities and underscored the importance of longitudinal monitoring of AMR profiles to understand the evolution of resistance genes. Although our study observed sparse AMR genes in this cohort of healthy Swiss children under 4 years of age, prior research indicates that the gut often harbors a higher abundance of ARGs in early life compared to adulthood (30, 31). This is likely due to early colonization by antibiotic-resistant bacteria from maternal and environmental sources, with ARG levels declining as the microbiome matures. To comprehensively understand AMR dynamics, longitudinal studies across diverse cohorts, including adults and elderly populations, are essential to capture age-specific patterns influenced by antibiotic exposure and microbiome development. Our study addressed a significant gap in the literature by demonstrating the feasibility of using home freezer storage for preserving stool samples. While different studies have investigated the temporal variability of a microbial community under different storage conditions, validating the stability of stool DNA stored in home freezers offers significant advantages for large-scale cohort studies, particularly those involving long-term follow-up and geographically dispersed participants. Notably, access to ultra-low temperature storage facilities (−80°C) is limited in many regions. By reducing the need for specialized storage facilities, researchers can optimize resource allocation and reduce logistical burdens on participants and study coordinators. This finding is particularly relevant for large-scale and citizen science projects, where cost-effective and accessible storage solutions are essential to promote inclusivity and broader participation in research (7).

Nevertheless, our study has several limitations. First, our study focused on a 6-month storage period. Future research should explore longer storage durations to assess longer-term storage effects. Additionally, our study included a relatively small sample size of 20 children, which may limit the generalizability of the results. A larger sample size would provide more robust data and allow for a more comprehensive analysis of variability across different individuals. While we employed ANCOM-BC to identify differentially abundant taxa at both the species and genus levels and observed no statistically significant differences over time, this approach relies on relative abundance data and does not capture absolute abundance. Future studies incorporating methods such as cell quantification (e.g., qPCR or flow cytometry) or spike-in standards during sequencing could provide deeper insights into temporal fluctuations in absolute abundance, which may reflect ecological dynamics not fully captured by the metrics used in this study. Future research should also include a broader range of sample types and participant cohorts such as individuals with gastrointestinal diseases (e.g., *Clostridioides difficile* infections) or those colonized by multiresistant organisms. These populations often harbor higher abundances and greater diversity of AMR genes due to increased antibiotic exposure and altered microbiomes, making them particularly relevant for assessing microbial composition and AMR gene stability under varying storage conditions. This will strengthen the applicability of findings for large-scale microbiome studies and clinical applications.

### Conclusion

The finding that home freezers can be used to effectively store stool samples for microbiome analysis significantly enhances the feasibility of long-term studies that involve at-home collection of stool samples. This approach promotes broader participation by allowing participants to conveniently store samples at home. Our results support the use of home freezer storage as a viable alternative to conventional methods, ensuring reliable and reproducible results. This advancement facilitates robust research into microbial dynamics and disease mechanisms. Future research should further explore the long-term stability of samples under various conditions to optimize preservation protocols and advance microbiome research.

## MATERIALS AND METHODS

### Stool sample collection and storage in home freezers

Fresh stool samples from 20 healthy Swiss children (55% female) with a mean age of 22.4 (range 2 to 56) months were aliquoted into four Fecon sterile tubes (Fecotainers, Medical Wire & Equipment Co. Ltd, United Kingdom). One aliquot was stored at 4°C and extracted and analyzed within 24 hours (0W), while the other three aliquots of the same sample were frozen in domestic freezers (below −18°C) and analyzed after 1 week (1W), 2 months (2M), and 6 months (6M). The domestic freezers used in this study included several models, such as V-ZUG Classic eco, Bosch Serie 6 NoFrost, Indesit Class TZAAA10.1, Liebherr MedLine, and Electrolux SG 164. The Bosch Serie 6 NoFrost and Liebherr MedLine freezers were frost-free models with automatic defrost cycles, while the V-ZUG, Indesit, and Electrolux models required manual defrosting. Samples were kept under the same conditions during transport to the Microbiota and Children Laboratory at the University of Fribourg, Switzerland.

### DNA extraction and metagenomic shotgun sequencing

Aliquots of stool samples were thawed at room temperature, and 100 mg of each sample was used for DNA extraction using the FastDNA SPIN Kit for Soil (MP Biomedicals, Illkirch-Graffenstaden, France) according to the manufacturer’s instructions. DNA concentrations were measured using Qubit dsDNA High Sensitivity Assay kits (Life Technologies, California, United States). The DNA in the negative control was below the detection limit (<0.01 ng/µL) and was not sequenced. Sequencing libraries with an insert size of approximately 600 bp were prepared using Nextera DNA Flex library preparation kits (Illumina, San Francisco, United States), with the addition of Illumina PhiX DNA. Paired-end sequencing (2  ×  149 bp) was performed on a NextSeq 550 system (Illumina) using high-output flow cells. Positive controls included bacterial and fungal mock communities (Gut Microbiome Whole Cell Mix MSA-2006 and Mycobiome Whole Cell Mix, respectively, ATCC, Manassas, United States).

### Bioinformatic analyses

#### Quality filtering and removal of human-mapping reads

FASTQC (v.0.11.9) was used to assess the quality of raw reads (32). Low-quality reads were filtered and trimmed using Trimmomatic v0.39 (33) with the following settings: LEADING:3, TRAILING:3, SLIDINGWINDOW:4:20, and MINLEN:36. Processed reads were then imported into QIIME 2 (Quantitative Insights Into Microbial Ecology 2)(34), and all further processing was performed using the MOSHPIT toolkit for shotgun metagenome analysis (35). Bowtie2 (36) and SAMtools (37) were used to align the reads against the human genome reference consortium (GRCh38) (38) to remove host sequences and retain nonhost (unmapped) reads for subsequent analysis, which resulted in a sum of 27,703,760 filtered reads across the data set.

### Microbiome taxonomy, diversity, and antimicrobial resistance gene profiling

The high-quality and filtered reads were taxonomically classified using Kraken2 (39) using the Standard database. This was followed by abundance re-estimation using Bracken (40) to enhance taxonomic profiling accuracy. Rarefied reads subsampled to the minimum sample read depth, in this case, 346,297 taxonomically annotated sequences per sample (excluding the unclassified portion), were compared on species-level dissimilarity based on beta diversity metrics, including Aitchison, Bray−Curtis, and Jaccard distances computed in QIIME 2. Additionally, alpha diversity metrics such as Shannon diversity and observed features (community richness) were also computed in QIIME 2.

To assess antimicrobial resistance (AMR) potential, reads were annotated using the Comprehensive Antibiotic Resistance Database (CARD) (41) in QIIME 2 using the q2-amr plugin (https://github.com/bokulich-lab/q2-amr). This involved using the Resistance Gene Identifier (RGI) application to predict antibiotic resistomes from protein or nucleotide data based on homology and single-nucleotide polymorphism (SNP) models. For validation using a database tailored to clinical applications, we used q2-amrfinderplus (https://github.com/bokulich-lab/q2-amrfinderplus) to annotate the assembled contigs with AMRFinderPlus (42).

### Metagenomic assembly and contig quality assessment

Trimmed and filtered reads from the individual samples were assembled into contigs using MEGAHIT (43) with default parameters using the QIIME 2 q2-assembly plugin (https://github.com/bokulich-lab/q2-assembly). Contig quality was assessed using metaQUAST (44) to calculate basic statistics such as assembly length, N50 values, and L50 metrics.

### Statistical analysis

Principal coordinate analysis (PCoA) was conducted using Aitchison, Bray−Curtis, and Jaccard distances in QIIME 2. To compare the mean Aitchison dissimilarity between different time points, the Wilcoxon rank-sum test was applied using the vegan package (v2.6–4) in RStudio (v2024.04.0 + 735), with *P*-values adjusted for multiple comparisons using the Bonferroni method. Additionally, PERMANOVA was performed with the vegan package (v2.6–4), utilizing the default number of permutations (*n* = 999). Pearson correlations were employed to assess the relationship between numerical or transformed metadata variables and principal components (PC1 and PC2) derived from the distance matrices. The Wilcoxon rank-sum test with Bonferroni correction was also used to evaluate differences in alpha diversity metrics, specifically Shannon diversity and observed features, across different time points. Differentially abundant analysis was performed at both species and genus levels using ANCOM-BC (45), with a minimum relative abundance of 1% across all samples.

To assess the influence of temporal versus inter-individual variation, we used the q2-sample-classifier plugin (46) (https://github.com/qiime2/q2-sample-classifier) in QIIME 2 to train a random forest classifier to predict sample time points based on stool microbiome profiles. The analysis employed standard parameters, including an 80/20 split for training and testing and 100 trees in the random forest model, with performance evaluated through stratified k-fold cross-validation to determine the model’s accuracy in predicting sample time points.

Linear mixed-effects (LME) models were employed to assess temporal trends in the microbial community composition and diversity while accounting for repeated measures within the same subjects over different time points and were performed using the QIIME 2 q2-longitudinal plugin (47) (https://github.com/qiime2/q2-longitudinal).

For constructing the heatmap and line-plots for antimicrobial resistance ontology (ARO) terms, normalized reads representing the number of sequence reads that were fully mapped to the reference sequence without any gaps were used to ensure accurate ARO term assignment. Mapped reads per sample were normalized for sequencing depth differences and to reduce sequencing bias by dividing the completely mapped reads by each sample’s total read count and multiplying by 1,000,000 to obtain reads per million (RPM). These RPM values were then log-transformed to account for the variation in read ranges. The top 50 most abundant ARO terms were plotted in a heatmap using ggplot2 (v3.5.1) in RStudio (v2024.04.0 + 735). Similarly, presence/absence heatmaps were generated for AMR-conferring genes identified with RGI and AMRFinderPlus. For RGI, the presence/absence was determined based on reads mapping 100% to the reference database, while for AMRFinderPlus, it was based on contigs with 100% mapping. To maintain consistency, the analysis was restricted to the top 50 most frequent AMR genes identified by each tool.

Statistical analysis based on the Wilcoxon rank-sum test with Bonferroni correction of the N50 and L50 metrics was conducted to assess the quality of the metagenomic assemblies. These metrics were calculated and compared across the different storage time points to evaluate any changes in assembly quality.

## Supplementary Material

Reviewer comments

## Data Availability

The sequencing raw data are deposited on the European Nucleotide Archive (ENA) with accession no. PRJEB79382. A STORMS (Strengthening The Organizing and Reporting of Microbiome Studies) checklist is available at DOI:10.5281/zenodo.13460391.

## SUPPLEMENTAL MATERIAL

Supplemental material spectrum.02278-24-s0001.docx Figures S1 to S4; Tables S1 to S3.

OPEN PEER REVIEW reviewer-comments.pdf An accounting of the reviewer comments and feedback.

ASM does not own the copyrights to Supplemental Material that may be linked to, or accessed through, an article. The authors have granted ASM a non-exclusive, world-wide license to publish the Supplemental Material files. Please contact the corresponding author directly for reuse.

## References

[1] Zimmermann P, Messina N, Mohn WW, Finlay BB, Curtis N. 2019. Association between the intestinal microbiota and allergic sensitization, eczema, and asthma: a systematic review. J Allergy Clin Immunol 143:467–485. doi:10.1016/j.jaci.2018.09.025 30600099

[2] Aldars-García L, Chaparro M, Gisbert JP. 2021. Systematic review: the gut microbiome and its potential clinical application in inflammatory bowel disease. Microorganisms 9:977. doi:10.3390/microorganisms9050977 33946482 PMC8147118

[3] Wang Y, Wei J, Zhang W, Doherty M, Zhang Y, Xie H, Li W, Wang N, Lei G, Zeng C. 2022. Gut dysbiosis in rheumatic diseases: a systematic review and meta-analysis of 92 observational studies. EBioMedicine 80:104055. doi:10.1016/j.ebiom.2022.104055 35594658 PMC9120231

[4] Górowska-Kowolik K, Chobot A. 2019. The role of gut micorbiome in obesity and diabetes. World J Pediatr 15:332–340. doi:10.1007/s12519-019-00267-x 31134588

[5] Nikolova VL, Smith MRB, Hall LJ, Cleare AJ, Stone JM, Young AH. 2021. Perturbations in gut microbiota composition in psychiatric disorders: a review and meta-analysis. JAMA Psychiatry 78:1343–1354. doi:10.1001/jamapsychiatry.2021.2573 34524405 PMC8444066

[6] Lauber CL, Zhou N, Gordon JI, Knight R, Fierer N. 2010. Effect of storage conditions on the assessment of bacterial community structure in soil and human-associated samples. FEMS Microbiol Lett 307:80–86. doi:10.1111/j.1574-6968.2010.01965.x 20412303 PMC3148093

[7] Vandeputte D, Tito RY, Vanleeuwen R, Falony G, Raes J. 2017. Practical considerations for large-scale gut microbiome studies. FEMS Microbiol Rev 41:S154–S167. doi:10.1093/femsre/fux027 28830090 PMC7207147

[8] Carroll IM, Ringel-Kulka T, Siddle JP, Klaenhammer TR, Ringel Y. 2012. Characterization of the fecal microbiota using high-throughput sequencing reveals a stable microbial community during storage. PLoS One 7:e46953. doi:10.1371/journal.pone.0046953 23071673 PMC3465312

[9] Roesch LFW, Casella G, Simell O, Krischer J, Wasserfall CH, Schatz D, Atkinson MA, Neu J, Triplett EW. 2009. Influence of fecal sample storage on bacterial community diversity. Open Microbiol J 3:40–46. doi:10.2174/1874285800903010040 19440250 PMC2681173

[10] Rius-Sansalvador B, Bars-Cortina D, Khannous-Lleiffe O, Serrano AG, Guinó E, Saus E, Gabaldón T, Moreno V, Obón-Santacana M. 2023. Stability of oral and fecal microbiome at room temperature: impact on diversity. bioRxiv. doi:10.1101/2023.11.28.568988 PMC12993597 41852417

[11] Liang Y, Dong T, Chen M, He L, Wang T, Liu X, Chang H, Mao J-H, Hang B, Snijders AM, Xia Y. 2020. Systematic analysis of impact of sampling regions and storage methods on fecal gut microbiome and metabolome profiles. mSphere 5:e00763-19. doi:10.1128/mSphere.00763-19 31915218 PMC6952195

[12] Nel Van Zyl K, Whitelaw AC, Newton-Foot M. 2020. The effect of storage conditions on microbial communities in stool. PLOS One 15:e0227486. doi:10.1371/journal.pone.0227486 31935223 PMC6959592

[13] Guo Y, Li S-H, Kuang Y-S, He J-R, Lu J-H, Luo B-J, Jiang F-J, Liu Y-Z, Papasian CJ, Xia H-M, Deng H-W, Qiu X. 2016. Effect of short-term room temperature storage on the microbial community in infant fecal samples. Sci Rep 6:26648. doi:10.1038/srep26648 27226242 PMC4880902

[14] Kim JH, Jeon J-Y, Im Y-J, Ha N, Kim J-K, Moon SJ, Kim M-G. 2023. Long-term taxonomic and functional stability of the gut microbiome from human fecal samples. Sci Rep 13:114. doi:10.1038/s41598-022-27033-w 36596832 PMC9810722

[15] Tedjo DI, Jonkers DMAE, Savelkoul PH, Masclee AA, van Best N, Pierik MJ, Penders J. 2015. The effect of sampling and storage on the fecal microbiota composition in healthy and diseased subjects. PLoS One 10:e0126685. doi:10.1371/journal.pone.0126685 26024217 PMC4449036

[16] Choo JM, Leong LEX, Rogers GB. 2015. Sample storage conditions significantly influence faecal microbiome profiles. Sci Rep 5:16350. doi:10.1038/srep16350 26572876 PMC4648095

[17] Wu GD, Lewis JD, Hoffmann C, Chen Y-Y, Knight R, Bittinger K, Hwang J, Chen J, Berkowsky R, Nessel L, Li H, Bushman FD. 2010. Sampling and pyrosequencing methods for characterizing bacterial communities in the human gut using 16S sequence tags. BMC Microbiol 10:206. doi:10.1186/1471-2180-10-206 20673359 PMC2921404

[18] Bassis CM, Moore NM, Lolans K, Seekatz AM, Weinstein RA, Young VB, Hayden MK, CDC Prevention Epicenters Program. 2017. Comparison of stool versus rectal swab samples and storage conditions on bacterial community profiles. BMC Microbiol 17:78. doi:10.1186/s12866-017-0983-9 28359329 PMC5374586

[19] Cardona S, Eck A, Cassellas M, Gallart M, Alastrue C, Dore J, Azpiroz F, Roca J, Guarner F, Manichanh C. 2012. Storage conditions of intestinal microbiota matter in metagenomic analysis. BMC Microbiol 12:158. doi:10.1186/1471-2180-12-158 22846661 PMC3489833

[20] Bokulich NA, Maldonado J, Kang D-W, Krajmalnik-Brown R, Caporaso JG. 2019. Rapidly processed stool swabs approximate stool microbiota profiles. mSphere 4:e00208-19. doi:10.1128/mSphere.00208-19 30971445 PMC6458435

[21] Gorzelak MA, Gill SK, Tasnim N, Ahmadi-Vand Z, Jay M, Gibson DL. 2015. Methods for improving human gut microbiome data by reducing variability through sample processing and storage of stool. PLOS One 10:e0134802. doi:10.1371/journal.pone.0134802 26252519 PMC4529225

[22] Song SJ, Amir A, Metcalf JL, Amato KR, Xu ZZ, Humphrey G, Knight R. 2016. Preservation methods differ in fecal microbiome stability, affecting suitability for field studies. mSystems 1:e00021-16. doi:10.1128/mSystems.00021-16 PMC5069758 27822526

[23] Zhang L, Fujinawa T, Saikawa M. 2016. Theoretical study on a frost-free household refrigerator-freezer. Int J Refrig 62:60–71. doi:10.1016/j.ijrefrig.2015.10.008

[24] Melo C, Knabben FT, Pereira PV. 2013. An experimental study on defrost heaters applied to frost-free household refrigerators. Appl Therm Eng 51:239–245. doi:10.1016/j.applthermaleng.2012.08.044

[25] Bansal P, Fothergill D, Fernandes R. 2010. Thermal analysis of the defrost cycle in a domestic freezer. Int J Refrig 33:589–599. doi:10.1016/j.ijrefrig.2009.11.012

[26] Ilett EE, Jørgensen M, Noguera-Julian M, Daugaard G, Murray DD, Helleberg M, Paredes R, Lundgren J, Sengeløv H, MacPherson C. 2019. Gut microbiome comparability of fresh-frozen versus stabilized-frozen samples from hospitalized patients using 16S rRNA gene and shotgun metagenomic sequencing. Sci Rep 9:13351. doi:10.1038/s41598-019-49956-7 31527823 PMC6746779

[27] Poulsen CS, Kaas RS, Aarestrup FM, Pamp SJ. 2021. Standard sample storage conditions have an impact on inferred microbiome composition and antimicrobial resistance patterns. Microbiol Spectr 9:e0138721. doi:10.1128/Spectrum.01387-21 34612701 PMC8510183

[28] Li X-M, Shi X, Yao Y, Shen Y-C, Wu X-L, Cai T, Liang L-X, Wang F. 2023. Effects of stool sample preservation methods on gut microbiota biodiversity: new original data and systematic review with meta-analysis. Microbiol Spectr 11:e0429722. doi:10.1128/spectrum.04297-22 37093040 PMC10269478

[29] Nagata N, Tohya M, Takeuchi F, Suda W, Nishijima S, Ohsugi M, Ueki K, Tsujimoto T, Nakamura T, Kawai T, Miyoshi-Akiyama T, Uemura N, Hattori M. 2019. Effects of storage temperature, storage time, and Cary-Blair transport medium on the stability of the gut microbiota. Drug Discov Ther 13:256–260. doi:10.5582/ddt.2019.01071 31611489

[30] Yassour M, Jason E, Hogstrom LJ, Arthur TD, Tripathi S, Siljander H, Selvenius J, Oikarinen S, Hyöty H, Virtanen SM, Ilonen J, Ferretti P, Pasolli E, Tett A, Asnicar F, Segata N, Vlamakis H, Lander ES, Huttenhower C, Knip M, Xavier RJ. 2018. Strain-level analysis of mother-to-child bacterial transmission during the first few months of life. Cell Host Microbe 24:146–154. doi:10.1016/j.chom.2018.06.007 30001517 PMC6091882

[31] Moore AM, Ahmadi S, Patel S, Gibson MK, Wang B, Ndao MI, Deych E, Shannon W, Tarr PI, Warner BB, Dantas G. 2015. Gut resistome development in healthy twin pairs in the first year of life. Microbiome 3:27. doi:10.1186/s40168-015-0090-9 26113976 PMC4480905

[32] Babraham Bioinformatics. 2024. FastQC a quality control tool for high throughput sequence data. Available from: https://www.bioinformatics.babraham.ac.uk/projects/fastqc. Retrieved 10 Jul 2024.

[33] Bolger AM, Lohse M, Usadel B. 2014. Trimmomatic: a flexible trimmer for Illumina sequence data. Bioinformatics 30:2114–2120. doi:10.1093/bioinformatics/btu170 24695404 PMC4103590

[34] Bolyen E, Rideout JR, Dillon MR, Bokulich NA, Abnet CC, Al-Ghalith GA, Alexander H, Alm EJ, Arumugam M, Asnicar F, et al.. 2019. Reproducible, interactive, scalable and extensible microbiome data science using QIIME 2. Nat Biotechnol 37:852–857. doi:10.1038/s41587-019-0209-9 31341288 PMC7015180

[35] Ziemski M, Gehret L, Simard A, Dau SC, Risch V, Grabocka D, Matzoros C, Wood C, Cabrera PM, Hernández-Velázquez R, Herman C, Evans K, Robeson MS, Bolyen E, Caporaso JG, Bokulich NA. 2025 MOSHPIT: accessible, reproducible metagenome data science on the QIIME 2 framework. Biorxiv. doi:10.1101/2025.01.27.635007

[36] Langmead B, Salzberg SL. 2012. Fast gapped-read alignment with Bowtie 2. Nat Methods 9:357–359. doi:10.1038/nmeth.1923 22388286 PMC3322381

[37] Li H, Handsaker B, Wysoker A, Fennell T, Ruan J, Homer N, Marth G, Abecasis G, Durbin R, 1000 Genome Project Data Processing Subgroup. 2009. The sequence alignment/map format and SAMtools. Bioinformatics 25:2078–2079. doi:10.1093/bioinformatics/btp352 19505943 PMC2723002

[38] Schneider VA, Graves-Lindsay T, Howe K, Bouk N, Chen H-C, Kitts PA, Murphy TD, Pruitt KD, Thibaud-Nissen F, Albracht D, et al.. 2017. Evaluation of GRCh38 and de novo haploid genome assemblies demonstrates the enduring quality of the reference assembly. Genome Res 27:849–864. doi:10.1101/gr.213611.116 28396521 PMC5411779

[39] Wood DE, Lu J, Langmead B. 2019. Improved metagenomic analysis with Kraken 2. Genome Biol 20:257. doi:10.1186/s13059-019-1891-0 31779668 PMC6883579

[40] Lu J, Breitwieser FP, Thielen P, Salzberg SL. 2017. Bracken: estimating species abundance in metagenomics data. PeerJ Comput Sci 3:e104. doi:10.7717/peerj-cs.104 PMC12016282 40271438

[41] Alcock BP, Huynh W, Chalil R, Smith KW, Raphenya AR, Wlodarski MA, Edalatmand A, Petkau A, Syed SA, Tsang KK, et al.. 2023. CARD 2023: expanded curation, support for machine learning, and resistome prediction at the Comprehensive Antibiotic Resistance Database. Nucleic Acids Res 51:D690–D699. doi:10.1093/nar/gkac920 36263822 PMC9825576

[42] Feldgarden M, Brover V, Gonzalez-Escalona N, Frye JG, Haendiges J, Haft DH, Hoffmann M, Pettengill JB, Prasad AB, Tillman GE, Tyson GH, Klimke W. 2021. AMRFinderPlus and the Reference Gene Catalog facilitate examination of the genomic links among antimicrobial resistance, stress response, and virulence. Sci Rep 11:12728. doi:10.1038/s41598-021-91456-0 34135355 PMC8208984

[43] Li D, Liu C-M, Luo R, Sadakane K, Lam T-W. 2015. MEGAHIT: an ultra-fast single-node solution for large and complex metagenomics assembly via succinct de Bruijn graph. Bioinformatics 31:1674–1676. doi:10.1093/bioinformatics/btv033 25609793

[44] Mikheenko A, Saveliev V, Gurevich A. 2016. MetaQUAST: evaluation of metagenome assemblies. Bioinformatics 32:1088–1090. doi:10.1093/bioinformatics/btv697 26614127

[45] Lin H, Peddada SD. 2020. Analysis of compositions of microbiomes with bias correction. Nat Commun 11:3514. doi:10.1038/s41467-020-17041-7 32665548 PMC7360769

[46] Bokulich NA, Dillon MR, Bolyen E, Kaehler BD, Huttley GA, Caporaso JG. 2018. Q2-sample-classifier: machine-learning tools for microbiome classification and regression. J Open Res Softw 3:934. doi:10.21105/joss.00934 31552137 PMC6759219

[47] Bokulich NA, Dillon MR, Zhang Y, Rideout JR, Bolyen E, Li H, Albert PS, Caporaso JG. 2018. q2-longitudinal: longitudinal and paired-sample analyses of microbiome data. mSystems 3:e00219-18. doi:10.1128/mSystems.00219-18 PMC6247016 30505944

