# Supplementary material for: Evaluating stool microbiome integrity after domestic freezer storage using whole-metagenome sequencing, genome assembly, and antimicrobial resistance gene analysis
Source: Microbiol Spectr. 2025 Feb 11;13(3):e02278-24. doi: 10.1128/spectrum.02278-24 (PMC11878046; doi:10.1128/spectrum.02278-24)

**Supplementary material**

## DNA concentration

## The mean DNA concentration per sample was 44.99 ng/µL (range 1.77 – 60 ng/µL). When analyzed on a per-child basis across all time points, some children, such as child S08, exhibited higher mean DNA concentrations with lower temporal variability, while others, like child S03, showed lower mean concentrations with higher temporal variability. Specifically, child S08 had a mean DNA concentration of 58.5 ng/µL (SD = 1.9 ng/µL), while child S03 displayed a mean concentration of 21.0 ng/µL (SD = 11.9 ng/µL), showing that individual children exhibited distinct patterns of DNA concentration measurements over time. However, Bonferroni-adjusted pairwise Wilcoxon tests among all children and time points revealed no significant differences in DNA concentrations.

**Figure S1.**  Principal Coordinates Analysis (PCoA) plot based on the **(a)** Bray-Curtis distance matrix and **(b)** Jaccard distance matrix illustrating the beta diversity of the microbial communities. Each dot represents a sample, colored by child ID (S01-S20).

**Table S1.** Linear Mixed Effects (LME) Model results for beta diversity distance metrics: Aitchison Distance, Bray-Curtis Distance, and Jaccard Distance. Coef. (Coefficient (β)), Std. Err. (Standard Error), t-value (t-statistic value), P>|t| (p-value associated with the t-statistic), and [95% Conf. Interval] (95% Confidence Interval for the coefficient estimates).

**Aitchison Distance**

**
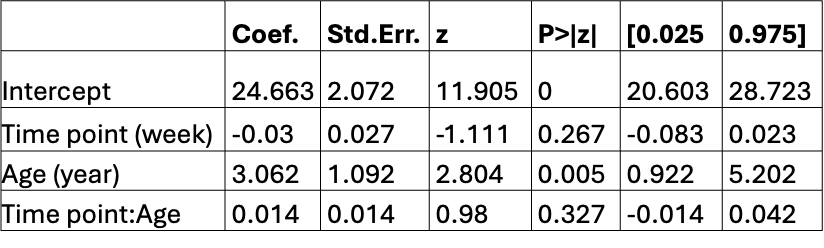
**

**Bray-Curtis Distance**

**
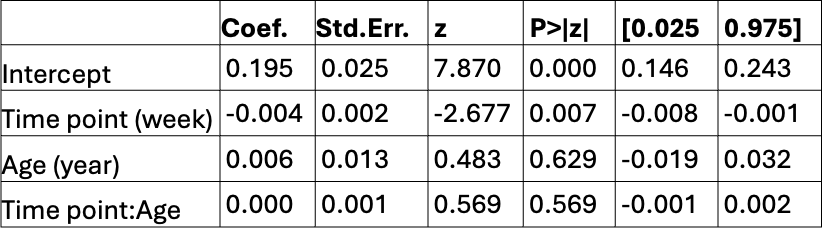
**

**Jaccard Distance**

**
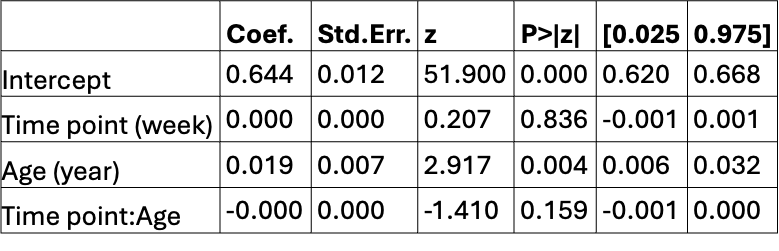
**

**Figure S2.** **(a)** Confusion matrix showing the Random Forest classifier's performance in predicting time points (0W = week 0, 1W = week 1, 2M = month 2, 6M = month 6) based on microbiome data. Rows represent true time points, and columns represent predicted time points, with color intensity indicating the proportion of samples classified. **(b)** ROC curves for each time point with corresponding AUC values. The dashed line represents random chance, with low AUC values indicating poor differentiation between time points based on microbiome profiles. **(c)** Summary of the model accuracy results, including overall accuracy, baseline accuracy, and accuracy ratio, demonstrating the model's performance across different time points.

**Figure S3.** Presence/absence heatmaps of the top 50 most frequent AMR-conferring genes identified using RGI and AMRFinderPlus. **(a)** AMR-conferring genes detected with RGI, based on reads mapping 100% to the reference database. **(b)** Heatmap of AMR-conferring genes detected with AMRFinderPlus, based on contigs mapping 100% to the reference database. Tiles represent the Present (blue) or Absent (white) genes in each sample. Samples are grouped by subject (S01–S20) and time point (0W = week 0, 1W = week 1, 2M = month 2, 6M = month 6) to visualize temporal trends in gene detection across the cohort.

**Figure S4.** Metadata variables contributing to PC1 and PC2 from PCoA of Jaccard distance of the AMR profiles at all four time points. **(a)** Pearson correlation coefficients were calculated between numeric metadata variables and principal coordinates (PC1 and PC2) to assess their linear relationships. **(b)** The bar plots show the metadata variables with the highest absolute correlation to the first (PC1) and second (PC2) principal components. Contributions are calculated as the absolute values of the correlations between metadata variables and principal coordinates.

**Table S2.** Permutational Multivariate Analysis of Variance (PERMANOVA) results for Aitchison, Bray-Curtis, and Jaccard distance matrices comparing the effects of week, subject, and age on microbial community composition. Df, Degrees of Freedom; SumOfSqs, Sum of Squares; R2, R-squared; F, F-statistic; Pr(>F), p-value; Perm, permutations.

**
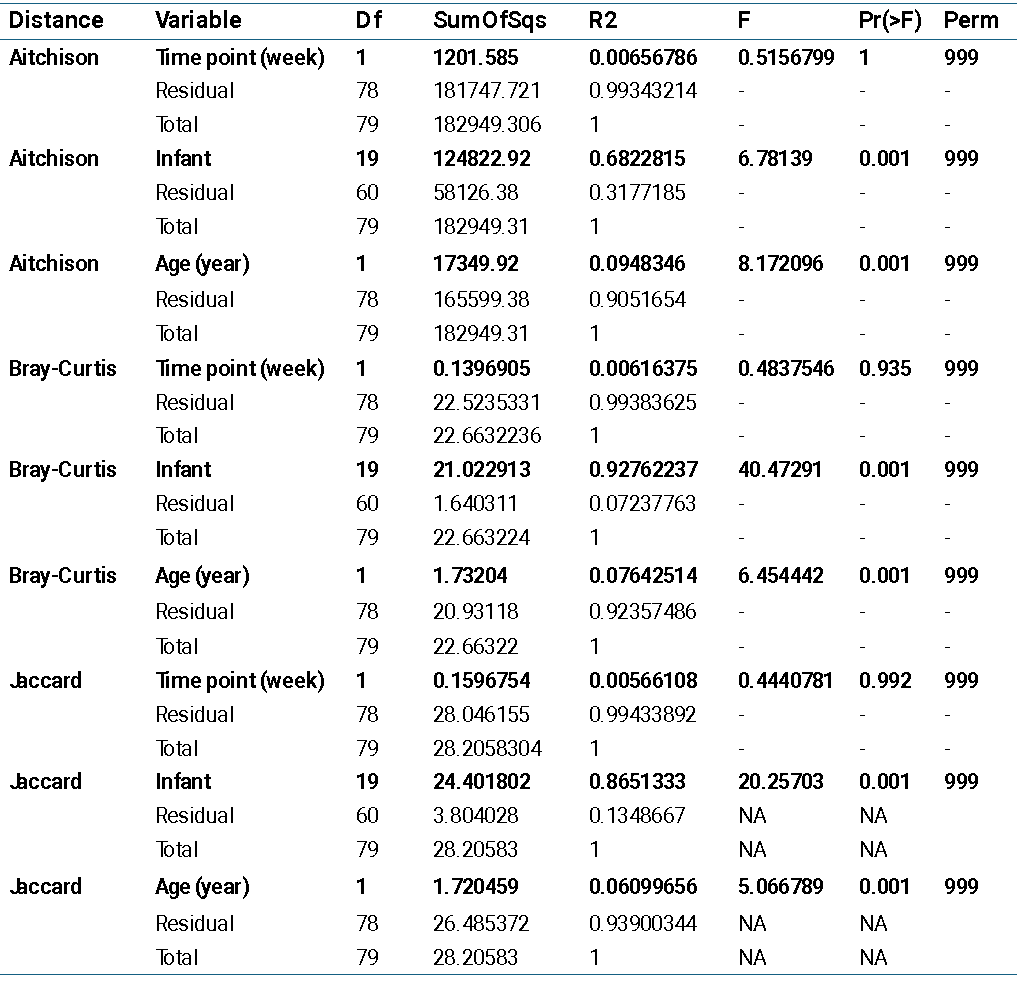
**

**Table S3.** Permutational Multivariate Analysis of Variance (PERMANOVA) results for the Jaccard distance matrix comparing the effects of week, subject, and age on AMR profile. Df, Degrees of Freedom; SumOfSqs, Sum of Squares; R2, R-squared; F, F-statistic; Pr(>F), p-value; Perm, permutations.


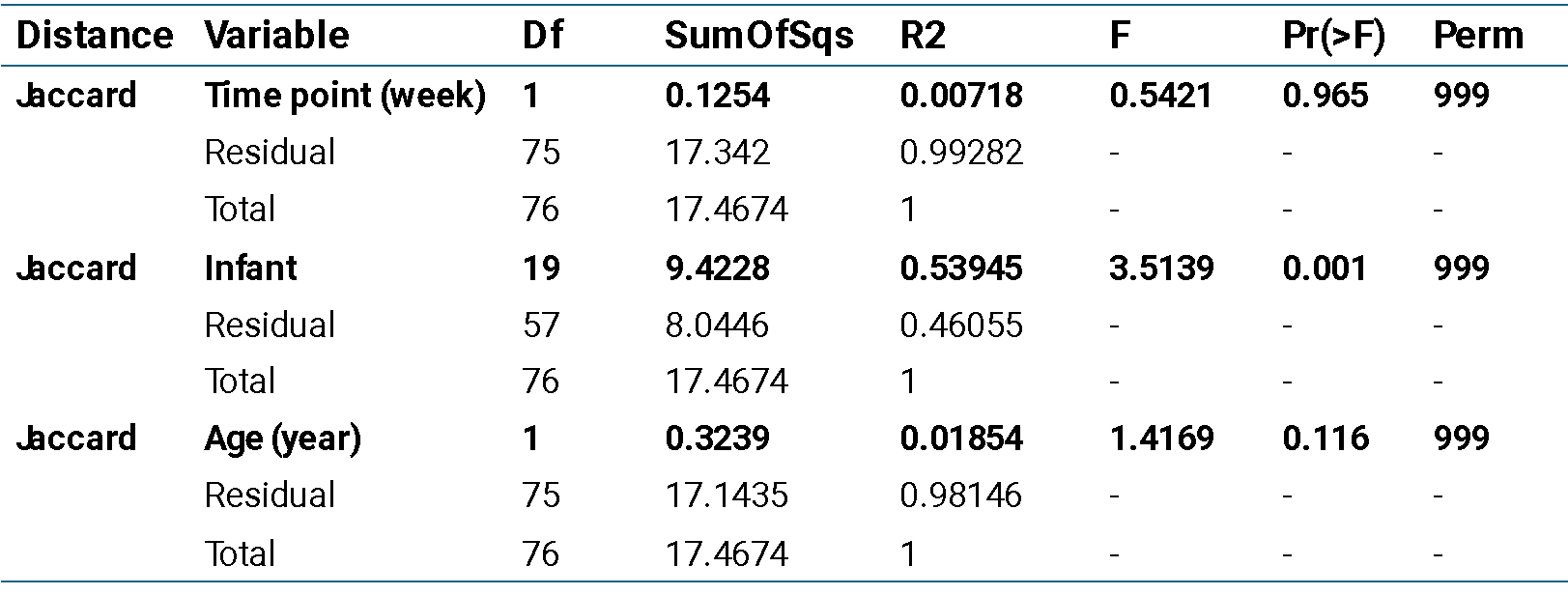

Supplement: Supplemental material — Figures S1 to S4; Tables S1 to S3. [file spectrum.02278-24-s0001.docx]
